# The effects of maternal cigarette smoking on cadmium and lead levels, miRNA expression and biochemical parameters across the feto-placental unit

**DOI:** 10.1016/j.heliyon.2022.e12568

**Published:** 2022-12-24

**Authors:** Ankica Sekovanić, Adrijana Dorotić, Daria Pašalić, Tatjana Orct, Zorana Kljaković-Gašpić, Antonija Sulimanec Grgec, Sandra Stasenko, Tatjana Mioč, Martina Piasek, Jasna Jurasović

**Affiliations:** a Analytical Toxicology and Mineral Metabolism Unit, Institute for Medical Research and Occupational Health, 10000 Zagreb, Croatia; b Department of Medical Laboratory Diagnostics, University Hospital Sveti Duh, 10000 Zagreb, Croatia; c Department of Medical Chemistry, Biochemistry and Clinical Chemistry, University of Zagreb, School of Medicine, 10000 Zagreb, Croatia; d Clinical Department of Obstetrics and Gynecology, Merkur University Hospital, 10000 Zagreb, Croatia

**Keywords:** miR-146a, miR-16, miR-21, Placenta, Postpartum women, Urate

## Abstract

Several miRNAs have been previously identified to be associated with cigarette smoke and/or the toxic metals cadmium (Cd) and lead (Pb). The aim of this study was to investigate the associations of maternal cigarette smoking with cadmium (Cd) and lead (Pb) levels, candidate miRNA expression and biochemical parameters across the feto-placental unit. miRNAs were isolated according to protocols provided by manufacturer from 72 healthy postpartum women using Qiagens’ kits based on phenol/guanidine samples lysis and silica-membrane purification of total RNA. Candidate miRNAs (miR-1537, miR-190b, miR-16, miR-21, and miR-146a) were quantified by real-time PCR. Biochemical parameters were analyzed in plasma samples by standardized and harmonized enzymatic methods using appropriate calibrators, while CRP was determined by immunoturbidimetric method. Concentration of Cd and Pb in whole blood and placenta samples were measured by inductively coupled plasma mass spectroscopy. Cd levels in smokers were higher in all of the analyzed compartments of the feto-placental unit, Pb in maternal blood and placenta than non-smokers. Smokers also had a higher expression of miR-16 in maternal and miR-146a in cord plasma, and lower expression of miR-21 in the placenta in comparison to non-smokers. Urate concentrations in the maternal plasma of smokers were lower than this value in non-smokers. The study has demonstrated that maternal smoking was associated with toxic metals (Cd and Pb) levels, urate concentration and alteration of miRNA expression. Given that the effects of maternal smoking on miRNA expression are inadequate, all compartments of the feto-placental unit should be analyzed to obtain a complete picture. This paper is the first to report on the results of expression of cellular and circulating miRNAs simultaneously in maternal and fetal compartments and in the placenta.

## Highlights

• Smokers have higher concentrations of Cd and Pb in maternal blood and placenta.
• Maternal smoking was associated with miRNA alteration across the feto-placental unit.
• Smokers had a higher expression of miR-16 in maternal and miR-146a in cord plasma.
• In placenta, lower expression of miR-21 was found in smokers.
• Decreased urate concentrations in maternal plasma were associated with smoking habit.

## Graphical abstract

**Figure:**
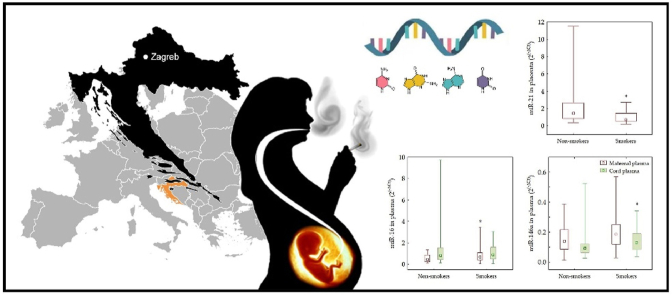

## 1 Introduction

The prenatal period of development is a critical period in the human lifetime when various exogenous factors, including maternal cigarette smoking, and endogenous factors, such as specific physiological state, may contribute to the developmental origins of health and disease (DOHaD) through the epigenetic regulation of gene expression. The effects of these factors are not evident in the primary nucleotide DNA sequence but can be reflected in changes to DNA methylation and microRNA (miRNA), small noncoding molecules able to control gene expression [1, 2, 3, 4, 5]. The potential of using miRNA expression as a biomarker of environmental exposure has recently been explored at an increasing rate [6]. However, this idea is still at a very early stage for a number of reasons, including diversity in routes and types of exposure, variability in study designs and detection methods and a lack of information on the biological relevance of miRNAs [7].

Maternal smoking habit during pregnancy has a well evidenced impact on wellbeing, reproductive outcomes and offspring development [8, 9, 10, 11, 12]. Cigarette smoke contains thousands of chemicals and it is well known that smoking habit is the main source of exposure to cadmium (Cd) and lead (Pb) in the general population [13, 14, 15]. Postpartum women who smoke have significantly higher levels of Cd and Pb in blood, placenta, and umbilical cord blood [16, 17]. During pregnancy, Cd accumulates in the placental tissue and placental Cd can be used as a valuable non-invasive biomarker of active maternal smoking [18, 19]. There is emerging evidence that maternal smoking habit and/or maternal exposure to metals from the environment may be associated with miRNA regulation [1, 2, 3, 4, 20]. However, studies that investigate the effects of exposure to toxic metals *in utero* through maternal cigarette smoking on miRNA regulation and the role that miRNAs have in the development of the disease in childhood and adulthood are lacking and inconsistent. Data report the downregulation of miR-16, miR-21 and miR-146a in the human placenta of smokers after vaginal delivery at term in comparison to non-smokers. Furthermore, high placental Pb levels are associated with decreased expression of several miRNAs including miR-146a, and miR-190b, while high placental Cd levels could have an impact on increased miR-1537 expression [20, 21]. Therefore, the aberrant repression of these miRNAs may have impact on the regulation of the cell cycle, immunomodulation and placental development, which can result in changes to fetal programming [1].

In addition to affecting the expression of miRNA, numerous toxic substances contained in cigarette smoke, including toxic metal ions, may also affect different biochemical indicators of the lipid metabolism, inflammation and oxidative stress. However, these associations have not been fully elucidated [22, 23]. Prospective studies have shown an association between maternal smoking habits and elevated total cholesterol concentrations and altered lipoprotein status in children [24]. There are findings that maternal smoking leads to metabolic changes in early fetal development and is associated with an increased risk of metabolic changes and impaired glucose homeostasis in adult offspring [25, 26]. Uric acid (urate), as a part of the body's antioxidant capacity, can also be altered by smoking. Previous studies have shown that plasma urate concentrations may be either lower [27] or higher [28, 29, 30] in smokers compared to non-smokers, but the reasons for the conflicting findings are still unclear. Also, there is evidence that children of smoking mothers may have a higher risk for oxidative stress, which may also be reflected in reduced urate concentrations [31].

Human epidemiological studies, which complement the results of experimental *in vivo* and *in vitro* studies, are necessary to observe the effects of different sources of environmental exposure on miRNAs in humans. Despite the evidence of long-term effects of maternal smoking during pregnancy on offspring health, few studies have explored the associations between maternal tobacco smoking during pregnancy, miRNA dysregulation and biochemical parameters. To address this gap in knowledge, this study evaluates the association of maternal cigarette smoking with toxic metals (Cd and Pb) levels, expression of candidate miRNAs (miR-1537, miR-190b, miR-16, miR-21, and miR-146a), and concentrations of selected biochemical parameters (C-reactive protein (CRP), glucose, urate, triglyceride, total cholesterol, HDL cholesterol, and LDL cholesterol) across the feto-placental unit.

## 2 Materials and methods

### 2.1 Study population and sample collection

This study included 72 mother-newborn pairs recruited during 2018 and 2019 at the Merkur University Hospital, Zagreb, Croatia within a wider national research project. The study participants were healthy postpartum women with normal vaginal birth at term (37^th^ – 42^th^ gestational week). The exclusion criteria in this study were medical history of pregnancy complications, fetal abnormalities and maternal chronic illness (diabetes mellitus, hypertension, eclampsia, peripheral edema). Participants were informed and familiar with the study aim and protocol, and they signed an informed consent form before inclusion in the study. Each participant was guaranteed anonymity by coding the samples and study data. The ethics committees of the collaborating institutions, Institute for Medical Research and Occupational Health, Merkur University Hospital, University of Zagreb School of Medicine, and University Hospital Sveti Duh in Zagreb reviewed and approved the study. All study protocols on the collection and use of personal data and biological samples comply with the principles of the Helsinki Declaration. Relevant data about medical history, personal data (age, education, body weight, parity), self-declared cigarette smoking (number of cigarettes smoked per day), and neonatal clinical data (gestational week, birth length and weight, APGAR scores) were collected and recorded by a questionnaire used and described in earlier studies conducted at the authors’ laboratory within the Institute for Medical Research and Occupational Health in Zagreb, Croatia [17, 32]. According to self-reported information about cigarette smoking habits, the study participants were divided into two groups using the following criteria: smokers – smoking any time during pregnancy or within 12 months before the last pregnancy, and non-smokers – never smoked or quit smoking more than 1 year before the pregnancy started.

Blood samples (maternal and cord blood) were collected in vacutainer tubes (Beckton-Dickinson, New Jersey, NJ, USA; with K_2_-EDTA anticoagulant). Maternal blood was collected before delivery, while cord blood and placenta were taken after childbirth in the maternity ward. Whole placentas were placed in clean zip-lock polyethylene bags and transferred to an analytical laboratory together with blood samples. Plasma samples were separated by centrifugation for 20 min at 3000 rpm (Hettich Rotanta/R, type 3501, Tuttlingen, Denmark), transferred into PCR clean polypropylene micro-tubes (Sarstedt, Nümbrecht, Germany) and stored at -80 °C until miRNA isolation. In the laboratory, placental tissue was sampled according to the study protocol within the shortest period possible following delivery (max 2 h). First, the rest of the umbilical cord was cut off and membranes trimmed, and then the placental disk was blotted on the absorbent mat and wet weight was measured. The decidua basalis and chorionic plate were cut off using a titanium knife, and the trophoblastic tissues were sampled as described elsewhere [17] and stored at -80 °C until the isolation of miRNAs.

### 2.2 miRNA isolation and reverse transcription to cDNA

Extracellular miRNAs from plasma samples were isolated using the miRNeasy Serum/Plasma Kit with miRNeasy Serum/Plasma Spike-in Control (miR-39 from *C. elegans*) and cellular miRNA from placenta samples using miRNeasy Mini Kit (Qiagen, Hilden, Germany) according to the method provided by the manufacturer. The concentrations of isolated mRNA enriched with miRNAs were checked spectrophotometrically using a NanoPhotometer P360 (Implen, München, Germany). Isolated miRNA was reverse transcribed to cDNA using miScript II RT Kit (Qiagen, Hilden, Germany) in 10 μL reaction volume for plasma (5 μL of isolated miRNA) and 20 μL for placenta (2 μL of isolated miRNA) under the following conditions: 1 h at 37 °C and 5 min at 95 °C on a Thermo-Mixer C (Eppendorf, Hamburg, Germany), and stored at -20 °C until pre-amplification step or analysis.

### 2.3 Pre-amplification of cDNA from plasma samples

Due to low amounts of extracellular miRNAs in human plasma, pre-amplification reactions were carried out in order to increase the amount of cDNA from plasma samples. Pre-amplification was performed using a miScript PreAMP PCR Kit (Qiagen, Hilden, Germany) according to the manufacturer's instructions in 12.5 μL reaction volume with primers mix (miR-16, miR-21, miR-146a, miR-190b, miR-1537 and control primers *C. elegans* miR-39, and miRTC) (Qiagen, Hilden, Germany) and diluted cDNA (1:4) as described in Sekovanić et al. [33]. The pre-amplification was done with 12 cycles (initial activation step 15 min at 95 °C and 2-step cycling: denaturation 30 s at 94 °C and annealing/extension 3 min at 60 °C) using a GeneAmp PCR System 2700 (Applied Biosystems, Waltham, MA, USA).

### 2.4 Real-time PCR quantification (qPCR)

Custom miScript miRNA PCR array with 96 wells and miScript SYBR Green PCR Kit (Qiagen, Hilden, Germany) were used to quantify candidate miRNAs (miR-1537, miR-190b, miR-16, miR-21, miR-146a) by qPCR. Preamplified cDNAs obtained from plasma samples were diluted 1:19 [33], while cDNA obtained from placenta samples were not preamplified and dilution was 1:10. The qPCR analysis was performed on an AB7500 (Applied Biosystems, Waltham, MA, USA) under the following conditions: initial activation step 15 min at 95 °C and 3-step cycling (denaturation 15 s at 94 °C, annealing 30 s at 55 °C and extension 30 s at 70 °C) in total of 40 cycles. Data were normalized using a cel-miR-39 for plasma and SNORD48 for placenta samples to minimize differences due to the quality of isolated miRNA and to obtain real changes in miRNA expression.

### 2.5 Cd and Pb analysis

Placenta samples (∼1 g) were weighed, and digested in nitric acid and ultrapure water (1:1) using UltraCLAVE IV (Milestone, Sorisole, Italy) microwave digestion system under the conditions presented in Table S1 (Supplementary Materials). Before analysis, placenta samples were diluted 20-fold with a solution containing 1% (v/v) HNO_3_ and 3 μg/L of internal standard (Ge, Rh, Lu, Tb and Ir), while maternal and cord blood was 70-fold diluted with a solution containing 0.7 mM ammonia, 0.01 mM EDTA, 0.07% (v/v) Triton X-100, and 3 μg/L of internal standard [17].

Concentrations of Cd and Pb in maternal and cord blood and placenta were determined by inductively coupled plasma–mass spectrometry (ICP-MS) on an Agilent 7500cx (Agilent Technologies, Tokyo, Japan). Before analysis, the instrument was optimized using a tune solution of 1 μg/L ^7^Li, ^59^Co, ^89^Y, ^140^Ce, and ^205^Tl and working conditions are shown in Table S2 (Supplementary Materials). The preparation and analysis of samples were carried out in a laboratory with a HVAC system (Heating, Ventilating and Air Conditioning) combined with HEPA filters. Commercially available reference materials for blood and tissue were used to check the accuracy of measurements, which was 90–110% for measured elements. The laboratory also regularly participates in the Interlaboratory Comparison Programme UK NEQAS (Birmingham, UK) for Trace Elements in Blood.

### 2.6 Biochemical parameters analysis

Biochemical parameters (CRP, glucose, urate, triglycerides, total cholesterol, HDL and LDL cholesterol) in maternal and cord plasma were determined with standardized and harmonized methods on an Atellica Solution (Siemens, Erlangen, Germany) according to the rules of good laboratory practice and recommendations from the Croatian Chamber of Medical Biochemists [34]. The methods used for biochemical analysis are shown in Table S3 (Supplementary materials) and were verified according to appropriate Clinical Laboratory Standards Institute guidelines. The reagents and calibrators were supplied by the analyzer manufacturer (Siemens, Erlangen, Germany) and the control samples for internal quality control were from Bio-Rad Laboratories (Hercules, California, USA).

### 2.7 Statistical analysis

Data are presented as the median (25–75 % interquartile range) and/or arithmetic mean with standard deviation (SD). Shapiro-Wilk test was used to test the normality of variables, and skewed data were logarithmically (ln) transformed before analysis. In microRNA analysis, C_t_ values ≤35.4 were considered as successful expressions, and all C_t_ values above the stated value were set to 36, i.e. as the critical number of cycles +1 [33]. The ΔC_t_ was calculated using equation C_t_-miRNA – C_t_-normalizer (where cel-miR-39 was normalizer for plasma samples and SNORD48 for placenta samples). Relative expression of analyzed miRNAs was calculated using the 2^(−ΔCt)^ method described in Pfaffl [35]. The results below LOD in element analysis were substituted with LOD/2 value [36], while in biochemical analysis these results were eliminated from further analysis. Mann-Whitney *U*-test was used to test the differences between smokers and non-smokers. Differences in miRNAs expression, toxic metal concentration and biochemical parameters between different compartments across the feto-placental unit were tested by Friedman test and *post-hoc* Wilcoxon matched-pairs test. Due to multiple *post-hoc* comparisons, we performed Bonferroni corrections, and for these analyses *p* < 0.017 was assigned as statistically significant. Multiple regression analysis was used to evaluate the possible influence of exposure to Cd and Pb via smoking on the relative miRNA expressions and levels of biochemical parameters in mother-newborn pairs. Models in multiple linear regression analysis include the following independent variables: maternal age, parity, weight gain during pregnancy, maternal education level, gestational week, placental weight, and Cd and Pb in maternal blood. Placental weight, and Cd and Pb in placenta were included only in models for placenta, while Cd and Pb in cord blood were included in models for cord blood The smoking status was not included in the regression models due to the strong correlation between the numbers of cigarettes smoked per day and Cd in maternal blood. Other confounding factors, such as passive smoke, diet, and environmental exposure, were not included in regression models duo to no differences were found between groups.

Statistical analysis were performed with software package TIBCO Statistica™, version 14.0.0.15 (TIBCO Software, Inc., Palo Alto, CA, USA). Statistical significance was set at 5% (*p* < 0.05) and all values below 0.05 were considered significant.

## 3 Results

### 3.1 General characteristics of the study group

The general characteristics of the study participants, mother-newborn pairs (n = 72), are shown in Table 1. The overall median of maternal years was 31 (range 21–44), whereas smokers were younger than non-smokers (29 *vs*. 33 years, *p* = 0.002). According to questionnaire data, 35 declared themselves as smokers with a median of 6.5 (2–20) cigarettes smoked per day (presented as median and min-max range). Despite public efforts to raise awareness of the harmfulness of smoking, women in Croatia and Europe smoke daily during pregnancy. In this study we found significant differences for education levels between smokers and non-smokers (*p* = 0.003). In addition, the percentage of women with a university degree in the non-smoker group was higher (56.8%) than in smokers (22.8%), where secondary school was dominant (68.6%). The average gestational time was 40 weeks and 60% of newborns were boys. All newborns were healthy with an average birth weight and length of 3,541 g and 51 cm, and the median APGAR score in the first and fifth minute was 10.

**Table 1:** General characteristics of the study participants (mother-newborn pairs) in relation to maternal smoking status.

	All (n = 72)	Non-smokers (n = 37)	Smokers (n = 35)	*p*
Maternal characteristics				
Age (years)	31 (21–44)	33 (21–44)	29 (21–41)	*0.002*
Education^1^				*0.003*
Primary school	5 (6.9)	2 (5.4)	3 (8.6)	
Secondary school	37 (51.4)	13 (35.1)	24 (68.6)	
University degree	29 (40.3)	21 (56.8)	8 (22.8)	
Body weight before pregnancy (kg)	65.7 ± 12.77	67.4 ± 15.49	63.9 ± 8.92	*0.243*
Body height (cm)	167 ± 5.7	168 ± 5.9	167 ± 5.6	*0.613*
Weight gain during pregnancy (kg)	14.2 ± 4.86	13.5 ± 4.75	14.9 ± 4.93	*0.202*
Gestation week at delivery	40 (36–42)	40 (37–42)	40 (36–42)	*0.960*
Trimmed placental weight (g)	411 ± 79.2	415 ± 84.5	406 ± 74.1	*0.627*
Parity	2 (1–6)	2 (1–6)	2 (1–6)	*0.620*
**Newborn characteristics**				
Girls	29 (40)	15 (40)	14 (40)	
Boys	43 (60)	22 (60)	21 (60)	
Birth weight (g)	3,541 ± 499	3,607 ± 513	3,471 ± 482	*0.251*
Birth length (cm)	51 ± 1.9	51 ± 1.9	51 ± 1.9	*0.480*
APGAR 1^st^ min score	10 (9–10)	10	10 (9–10)	*0.307*
APGAR 5^th^ min score	10	10	10	

Results are presented as mean ± SD, median (min-max) or number and percentage (%).

Differences between smokers and non-smokers tested by Student's *t*-test or Fisher's test (*p* < 0.05).

1 One participant did not report her education level.

### 3.2 Comparison of analyzed parameters between smokers and non-smokers

#### 3.2.1 Cd and Pb concentration

Concentrations of Cd and Pb in maternal blood, placenta and cord blood related to maternal smoking status are shown in Table 2. Smokers had 2.5-fold higher levels of Cd in maternal blood, and 1.5-fold in placenta and cord blood in comparison to non-smokers (*p* < 0.02). Pb concentrations in smokers were 1.5-fold higher in maternal blood (*p* = 0.022) and placenta (*p* = 0.002), while no significant differences in cord blood (*p* = 0.061) in comparison to non-smokers. Table 3 shows Spearman's correlation coefficients between number of cigarettes smoked per day and Cd and Pb levels. Medium correlation was found between the number of cigarettes smoked per day and Cd levels in maternal blood (ρ_s_ = 0.683), while correlation between number of cigarettes smoked per day and Cd in placenta and cord blood was weak (ρ_s_ = 0.327–0.380), as well as between number of cigarettes and Pb in placenta (ρ_s_ = 0.382).

**Table 2:** Cadmium (Cd) and lead (Pb) levels in mother-newborn pairs related to maternal smoking status.

	Non-smokers (n = 37)	Smokers (n = 35)	*p*
Cd in maternal blood (μg/L)	0.261 (0.212–0.344)	0.589 (0.400–1.13)	*<0.001*
Cd in placenta (μg/kg)	5.48 (4.39–7.34)	7.62 (5.28–10.5)	*0.014*
Cd in cord blood (μg/L)	0.016 (0.008–0.027)	0.029 (0.020–0.035)	*0.009*
Pb in maternal blood (μg/L)	7.54 (5.67–10.9)	9.59 (8.08–11.4)	*0.022*
Pb in placenta (μg/kg)	1.44 (1.06–2.52)	2.56 (1.86–3.66)	*0.002*
Pb in cord blood (μg/L)	5.75 (3.92–7.33)	6.60 (5.09–8.14)	*0.061*

Results are presented as median (25–75% interquartile range).

Differences between smokers and non-smokers tested by Mann-Whitney *U*-test (*p* < 0.05).

**Table 3:** Spearman's correlation coefficients (ρ_s_) for relationships between number of cigarettes smoked per day and cadmium (Cd) and lead (Pb) in all measured samples.

	Cd in maternal blood	Cd in placenta	Cd in cord blood	Pb in maternal blood	Pb in placenta	Pb in cord blood
Cigarettes per day	**0.683**	**0.327**	**0.380**	0.198	**0.382**	0.190
Cd in maternal blood		**0.710**	**0.437**	**0.270**	**0.351**	0.201
Cd in placenta			**0.328**	**0.270**	**0.308**	**0.282**
Cd in cord blood				0.147	0.192	**0.237**
Pb in maternal blood					**0.535**	**0.822**
Pb in placenta						**0.586**

Significant correlations (*p* < 0.05) are marked in bold.

#### 3.2.2 miRNAs expression

**Figure 1:**
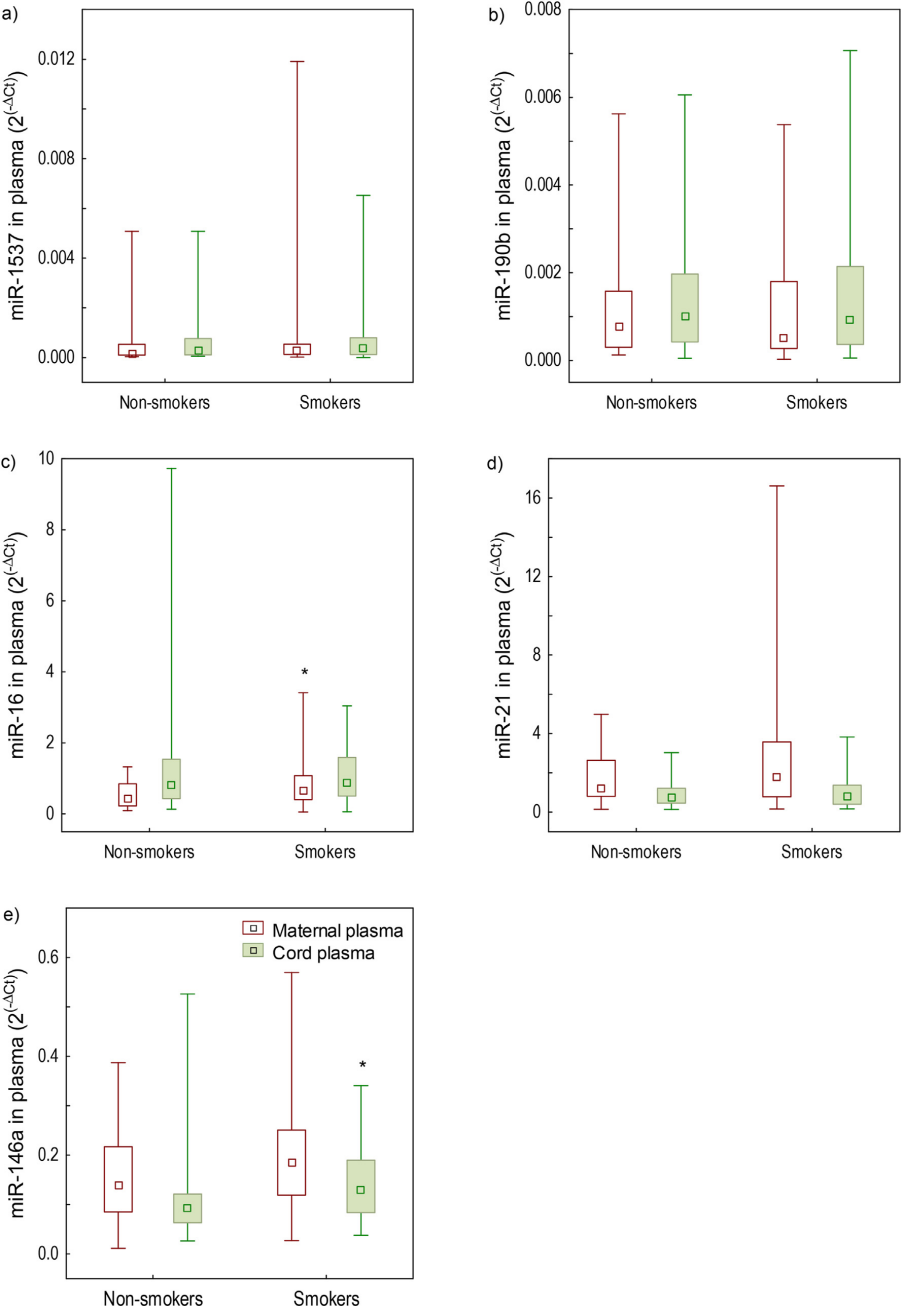
miRNA relative expressions (2^(−ΔCt)^) of: a) miR-1537, b) miR-190b, c) miR-16, d) miR-21, e) miR-146a in maternal () and umbilical cord () plasma determined in the study of mother-newborn pairs from Croatia (n = 65). Data are presented by Box-and-Whisker plots where boundaries of box-plots indicate the 25^th^ and 75^th^ percentiles; the square within the box is the median value; whiskers above and below the box indicate the minimum and maximum. ΔCt was calculated using equation Ct-miRNA – Ct-normalizer (where cel-miR-39 was normalizer for plasma samples and SNORD48 for placenta samples). ∗Differences between smokers and nonsmokers were tested by Mann-Whitney *U*-test (*p* < 0.05).

**Figure 2:**
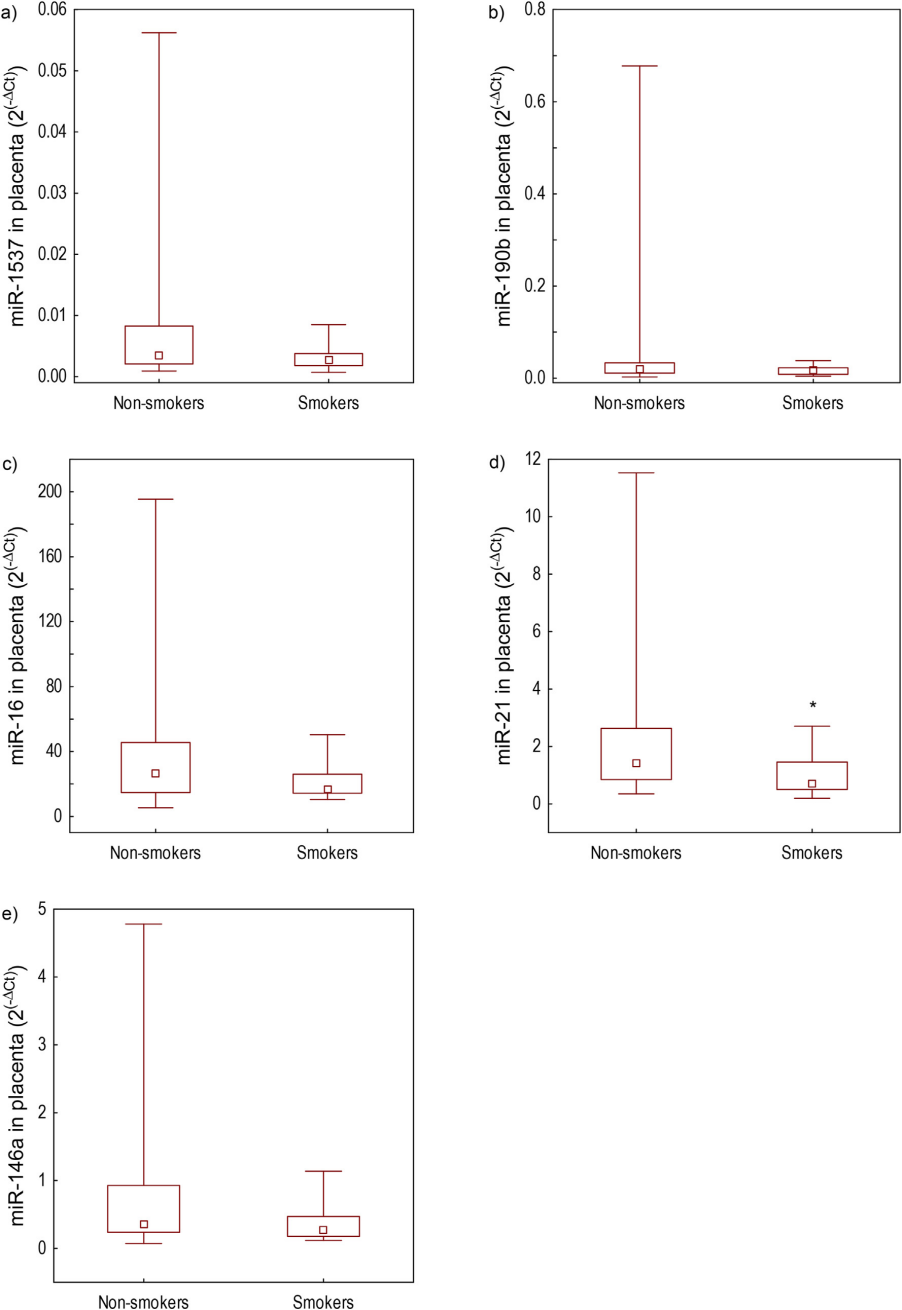
miRNA relative expressions (2^(−ΔCt)^) of: a) miR-1537, b) miR-190b, c) miR-16, d) miR-21, e) miR-146a in placenta of smokers and non-smokers determined in the study of mother-newborn pairs from Croatia (n = 72). Data are presented by Box-and-Whisker plots where boundaries of box-plots indicate the 25^th^ and 75^th^ percentiles; the square within the box is the median value; whiskers above and below the box indicate minimum and maximum. ΔCt was calculated using equation Ct-miRNA – Ct-normalizer (where cel-miR-39 was normalizer for plasma samples and SNORD48 for placenta samples). ∗Differences between smokers and non-smokers were tested by Mann-Whitney *U*-test (*p* < 0.05).

Relative expressions of candidate miRNAs in maternal and cord plasma and placenta in relation to maternal smoking are shown on Figures 1 and 2. Higher expressions of miR-16 in maternal plasma (*p* = 0.037) and miR-146a in cord plasma (*p* = 0.031) were found in smokers *vs*. non-smokers. Furthermore, expression of miR-21 in placenta was lower in smokers than non-smokers (*p* < 0.001). No differences between smokers and non-smokers were found for the other analyzed miRNAs.

#### 3.2.3 Biochemical parameters

Table 4 presents the biochemical parameters obtained in the maternal and cord plasma of non-smokers and smokers. A decreased concentration of urate in maternal plasma was found in smokers in comparison to non-smokers (*p* = 0.032), while no differences in the other measured parameters were found between the groups. Concentrations of CRP in cord plasma were below the limit of detection, while a concentration of HDL cholesterol in cord plasma was detected in only 40 samples (19 non-smokers and 21 smokers).

**Table 4:** Biochemical parameters in the maternal and umbilical cord plasma of smokers and non-smokers.

	Non-smokers (n = 30)	Smokers (n = 35)	*p*
Glucose in maternal plasma (mmol/L)	5.6 (5.1–7.3)	5.7 (4.9–6.7)	*0.465*
Glucose in cord plasma (mmol/L)	4.2 (3.7–4.9)	4.3 (3.7–4.9)	*0.813*
Triglycerides in maternal plasma (mmol/L)	3.0 (2.2–3.5)	2.9 (2.0–3.6)	*0.921*
Triglycerides in cord plasma (mmol/L)	0.46 (0.41–0.52)	0.49 (0.41–0.55)	*0.338*
Urate in maternal plasma (μmol/L)	295 (263–323)	255 (227–302)	*0.032*
Urate in cord plasma (μmol/L)	296 (244–310)	255 (227–311)	*0.129*
Total cholesterol in maternal plasma (mmol/L)	6.4 (6.1–7.6)	6.5 (6.0–7.2)	*0.906*
Total cholesterol in cord plasma (mmol/L)	1.6 (1.3–1.8)	1.6 (1.4–1.8)	*0.829*
HDL in maternal plasma (mmol/L)	2.0 (1.5–2.2)	1.8 (1.4–2.1)	*0.614*
HDL in cord plasma (mmol/L)#	0.67 (0.57–0.85)	0.62 (0.56–0.68)	*0.228*
LDL in maternal plasma (mmol/L)	4.2 (3.4–4.9)	4.4 (3.5–4.9)	*0.833*
LDL in cord plasma (mmol/L)	0.46 (0.35–0.57)	0.50 (0.36–0.67)	*0.479*
CRP in maternal plasma (mg/l)	7.9 (3.4–16.2)	8.2 (5.3–13.1)	*0.652*
CRP in cord plasma (mg/L)	<LOD	<LOD	

Results are presented as median (25–75% interquartile range).

Differences between smokers and non-smokers tested by Mann-Whitney *U*-test (*p* < 0.05).

LOD: limit of detection (LOD = 0.5 mg/L).

# Detected in 19 non-smokers and 21 smokers, other values were below the LOD (LOD = 0.52 mmol/L).

### 3.3 Comparison of analyzed parameters across the feto-placental unit

#### 3.3.1 Cd and Pb concentration

The highest concentration of Cd was found in placenta (overall median 6.3; IQR 4.9–8.8 μg/kg) and the lowest in cord blood (overall median 0.02; IQR 0.01–0.03 μg/L). Cd levels in maternal blood were 10- to 20-fold lower than values in placenta (overall median 0.34; IQR 0.25–0.69 μg/L). Unlike Cd, the Pb concentration was highest in maternal blood (overall median 8.6; IQR 6.9–11.0 μg/L) and lowest in placenta (overall median 2.1; IQR 1.3–3.4 μg/kg). Pb levels measured in cord blood were 69–76% of the levels in maternal blood (overall median 6.3; IQR 4.3–7.7 μg/L) (Table 2). A strong correlation was found between Cd in maternal blood and Cd in placenta (ρ_s_ = 0.710), and between Pb in maternal blood and Pb in cord blood (ρ_s_ = 0.822). A medium correlation was found between Pb in maternal blood and Pb in placenta (ρ_s_ = 0.535), and Pb in placenta and Pb in cord blood (ρ_s_ = 0.586). Furthermore, Cd in maternal blood was weakly correlated with Cd in cord blood (ρ_s_ = 0.437), as well as Cd in placenta with Cd in cord blood (ρ_s_ = 0.328) (Table 3).

#### 3.3.2 miRNAs expression

The relative expression of each analyzed miRNA (miR-1535, miR-190b, miR-16, miR-21 and miR-146a) showed a statistically significant difference between compartments of the feto-placental unit, regardless of whether we compared them in smokers or non-smokers (Table 5). The highest expressions of miR-1537, miR-190b, miR-16 and miR-146a were found in the placenta, being significantly higher than in maternal or cord plasma (*p* < 0.001). There was no statistical significance in expressions of miR-1537 and miR-190b between maternal and cord plasma of smokers or non-smokers, or for miR-16 in smokers. However, differences in miR-16 expression in maternal and cord plasma of non-smokers were statistically significant, with higher expression in cord plasma (*p* = 0.001). Expression of miR-146a was significantly higher in maternal plasma than in cord plasma of smokers (*p* = 0.006). Although the highest expression of miR-21 was found in the placenta of non-smokers, no statistical differences were found between placenta and maternal or cord plasma. In contrast to non-smokers, smokers had the highest miR-21 expression in maternal plasma, with a statistically significant difference between maternal plasma and the placenta (*p* < 0.001).

**Table 5:** miRNA relative expressions (2^(−ΔCt)^) in mother-newborn pairs related to maternal smoking status.

	Non-smokers (n = 37)			Smokers (n = 35)		
	Median (IQR)	Friedman test *p*-value	Wilcoxon matched-pairs test *p*-value	Median (IQR)	Friedman test *p*-value	Wilcoxon matched-pairs test *p*-value
miR-1537 in maternal plasma	0.0002 (0.0001–0.0005)	<0.001	MP/CP = 0.239	0.0003 (0.0001–0.0005)	<0.001	MP/CP = 0.437
miR-1537 in placenta	0.0036 (0.0021–0.0083)		MP/PL < 0.001	0.0027 (0.0018–0.0038)		MP/PL < 0.001
miR-1537 in cord plasma	0.0003 (0.0001–0.0008)		PL/CP < 0.001	0.0004 (0.0001–0.0008)		PL/CP < 0.001
miR-190b in maternal plasma	0.0008 (0.0003–0.0016)	<0.001	MP/CP = 0.441	0.0005 (0.0003–0.0018)	<0.001	MP/CP = 0.228
miR-190b in placenta	0.0196 (0.0112–0.0333)		MP/PL < 0.001	0.0164 (0.0085–0.0226)		MP/PL < 0.001
miR-190b in cord plasma	0.0010 (0.0004–0.0020)		PL/CP < 0.001	0.0009 (0.0004–0.0021)		PL/CP < 0.001
miR-16 in maternal plasma	0.4378 (0.2274–0.8478)	<0.001	MP/CP = 0.001	0.6664 (0.4035–1.076)∗	<0.001	MP/CP = 0.159
miR-16 in placenta	26.33 (14.70–45.51)		MP/PL < 0.001	16.84 (14.27–25.97)		MP/PL < 0.001
miR-16 in cord plasma	0.8167 (0.4321–1.538)		PL/CP < 0.001	0.8729 (0.5031–1.588)		PL/CP < 0.001
miR-21 in maternal plasma	1.186 (0.8004–2.640)	<0.001	MP/CP = 0.003	1.796 (0.7841–3.574)	0.002	MP/CP < 0.001
miR-21 in placenta	1.415 (0.8488–2.632)		MP/PL = 0.569	0.6872 (0.5022–1.458)∗		MP/PL < 0.001
miR-21 in cord plasma	0.7483 (0.4524–1.209)		PL/CP = 0.053	0.7952 (0.4044–1.364)		PL/CP = 0.875
miR-146a in maternal plasma	0.1397 (0.0852–0.2170)	<0.001	MP/CP = 0.033	0.1855 (0.1191–0.2508)	<0.001	MP/CP = 0.006
miR-146a in placenta	0.3617 (0.2361–0.9286)		MP/PL < 0.001	0.2719 (0.1770–0.4706)		MP/PL < 0.001
miR-146a in cord plasma	0.0917 (0.0633–0.1212)		PL/CP < 0.001	0.1297 (0.0836–0.1899)∗		PL/CP < 0.001

Results are presented as median (25–75% interquartile range). ΔC_t_ was calculated using equation C_t_-miRNA – C_t_-normalizer (where cel-miR-39 was normalizer for plasma samples and SNORD48 for placenta samples).

Differences between different compartments across the feto-placental unit were tested by Friedman test and *post-hoc* Wilcoxon matched-pairs test (*p* < 0.017).

MP: maternal plasma; CP: umbilical cord plasma PL: placenta.

∗ Significant differences between smokers and non-smokers tested by Mann-Whitney *U*-test (*p* < 0.05).

#### 3.3.3 Biochemical parameters

Concentrations of triglycerides in maternal plasma were 6-fold higher (overall median 2.9; IQR 2.1–3.5 mmol/L) than in cord plasma (overall median 0.46; IQR 0.41–0.54 mmol/L). Concentrations of glucose in cord plasma were 75% of the values in maternal plasma. In addition, the concentrations of total cholesterol, HDL and LDL cholesterol were higher in maternal plasma than in cord plasma. No difference between maternal and cord plasma was found for urates (Table 4).

### 3.4 Association between Cd and Pb with miRNAs expression, biochemical parameters and birth outcomes

Tables 6 and 7 show Spearman's correlation coefficients for the relationships between maternal smoking habit (number of cigarettes smoked per day) and concentrations of Cd and Pb in maternal blood, placenta and cord blood and relative expressions of candidate miRNAs (Table 6) or the measured biochemical parameters (Table 7). The miR-16 in maternal plasma was positively, although weakly, correlated with cigarette number (ρ_s_ = 0.302), as well as with Cd and Pb in all three compartments of the maternal-placental-fetal unit (ρ_s_ = 0.265–0.357). A weak positive correlation was also found between Cd and miR-16 in cord plasma (ρ_s_ = 0.312). Negative weak correlations were found between the number of smoked cigarettes and miR-21 in placenta (ρ_s_ = -0.348), Cd in maternal blood and miR-1537 in maternal (ρ_s_ = -0.263) and miR-190b in cord (ρ_s_ = -0.286) plasma, and between Cd in placenta and miR-21 in maternal (ρ_s_ = -0.252) and miR-190b in cord (ρ_s_ = -0.274) plasma.

**Table 6:** Spearman's correlation coefficients (ρ_s_) for relationships between smoking habit (number of cigarettes smoked per day), biomarkers of cadmium (Cd) and lead (Pb), and relative expressions of candidate miRNAs determined in the mother-newborn pairs from Croatia (n = 72).

	miR-1537_MP	miR-1537_PL	miR-1537_CP	miR-190b_MP	miR-190b_PL	miR-190b_CP	miR-16_ MP	miR-16_ PL	miR-16_ CP	miR-21_ MP	miR-21_ PL	miR-21_ CP	miR-146a_MP	miR-146a_PL	miR-146a_CP
Cigarettes per day	0.001	-0.175	0.029	-0.058	-0.202	-0.137	**0.302**	-0.168	0.106	-0.021	**-0.348**	-0.083	0.112	-0.135	0.172
Cd_MB	**-0.263**	-0.159	-0.061	-0.121	-0.110	**-0.286**	**0.357**	-0.110	0.007	-0.167	-0.182	-0.161	0.106	0.060	0.153
Cd_PL	-0.239	-0.034	-0.147	-0.198	0.009	**-0.274**	**0.277**	0.038	0.030	**-0.252**	-0.080	-0.061	0.098	0.043	0.238
Cd_CB	-0.152	-0.140	0.056	-0.238	-0.093	-0.051	**0.344**	-0.057	**0.312**	-0.157	-0.066	-0.012	0.128	0.112	0.184
Pb_MB	0.124	-0.018	0.018	0.002	-0.033	0.105	**0.278**	-0.099	0.067	0.146	-0.159	0.061	0.227	-0.036	0.104
Pb_PL	-0.036	-0.101	-0.063	-0.109	0.012	-0.133	**0.265**	0.017	0.203	-0.094	-0.108	-0.099	0.053	-0.018	0.114
Pb_CB	0.170	-0.093	0.021	-0.018	-0.102	0.075	**0.292**	-0.060	0.062	0.138	-0.218	-0.032	0.216	-0.086	0.053

MB: maternal blood; PL: placenta; CB: umbilical cord blood; MP: maternal plasma; CP: umbilical cord plasma. Significant correlations (*p* < 0.05) are marked in bold.

**Table 7:** Spearman's correlation coefficients (ρ_s_) for relationships between smoking habit (number of cigarettes smoked per day), biomarkers of cadmium (Cd) and lead (Pb), and biochemical parameters determined in the mother-newborn pairs from Croatia (n = 65).

	TC_ MP	TC_ CP	HDL_MP	HDL_CP	LDL_MP	LDL_CP	GLU_MP	GLU_CP	TG_MP	TG_ CP	Urate_MP	Urate_CP	CRP_MP
Cigarettes per day	-0.014	0.059	-0.080	-0.229	0.041	0.118	-0.140	-0.069	-0.066	0.184	-0.174	-0.095	0.119
Cd_MB	0.113	0.236	0.067	0.045	0.129	0.174	-0.163	-0.043	0.058	0.133	-0.125	-0.050	0.091
Cd_PL	0.136	0.185	0.060	0.128	0.114	0.155	-0.155	-0.062	0.208	-0.054	0.085	0.085	0.187
Cd_CB	**0.249**	0.209	0.123	-0.053	**0.256**	0.153	0.038	-0.078	0.029	0.127	-0.208	-0.181	0.044
Pb_MB	**0.323**	0.145	0.125	0.125	**0.290**	0.064	0.098	-0.018	0.186	0.098	0.001	0.017	-0.114
Pb_PL	0.164	0.060	0.082	-0.235	0.164	0.115	0.029	0.023	0.008	0.244	-0.140	-0.137	0.079
Pb_CB	0.205	0.191	0.031	0.101	0.240	0.173	0.151	0.003	0.166	0.129	0.051	0.050	0.049

MB: maternal blood; PL: placenta; CB: umbilical cord blood; MP: maternal plasma; CP: umbilical cord plasma; TC: total cholesterol; HDL: high-density lipoprotein; LDL: low-density lipoprotein; GLU: Glucose; TG: triglycerides; CRP: C-reactive protein. Significant correlations (*p* < 0.05) are marked in bold.

Total cholesterol and LDL cholesterol in maternal plasma were weakly correlated with Cd in cord blood (ρ_s_ of 0.249 and 0.256, respectively) and Pb in maternal blood (ρ_s_ of 0.323 and 0.290, respectively) (Table 7).

**Figure 3:**
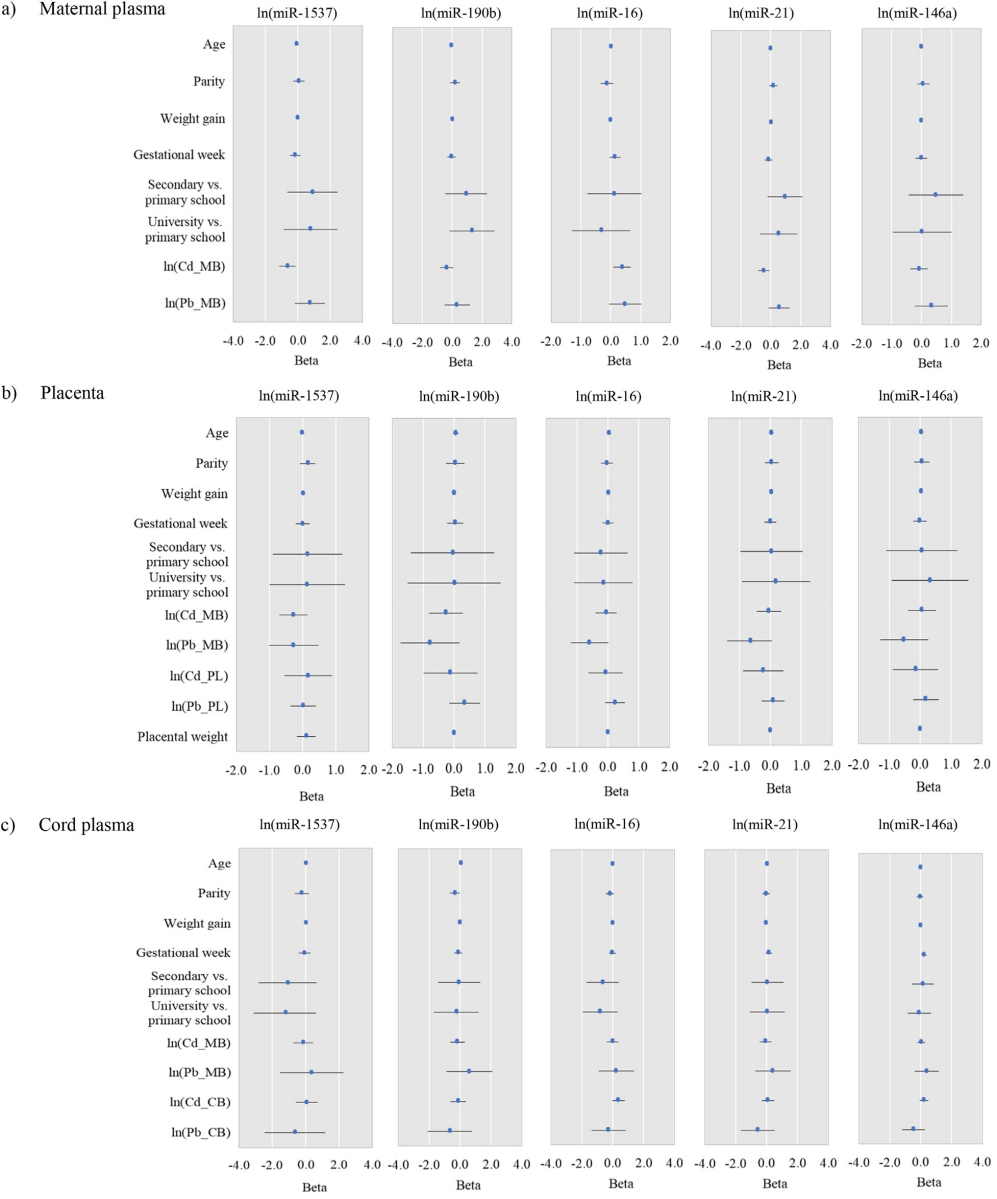
Results of multiple regression analysis for relative expression (2^(−ΔCt)^) of circulating candidate microRNAs (miR-1537, miR-190b, miR-16, miR-21 and miR-146a) in: a) maternal plasma, b) placenta, c) cord plasma. Data are presented as beta and 95% confidence intervals. Models included the following variables: maternal age, parity, weight gain during pregnancy, maternal education level, gestational week, and Cd and Pb in maternal blood. Placental weight, and Cd and Pb in placenta were included only in models for placenta, while Cd and Pb in cord blood were included in models for cord blood. The smoking status was not include due to the strong correlation between the numbers of cigarettes smoked per day and Cd in maternal blood. Skewed data were normalized using logarithmic (ln) transformation before analysis. MB: maternal blood; PL: placenta; CB: umbilical cord blood.

**Figure 4:**
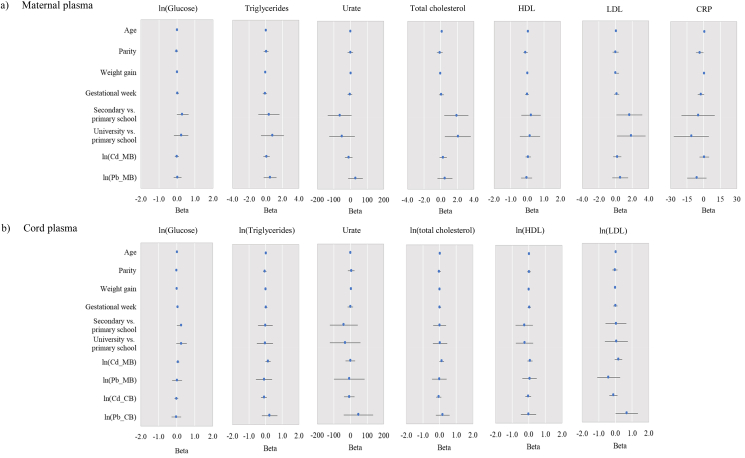
Results of multiple regression analysis for biochemical parameters (glucose, triglycerides, urate, total cholesterol, HDL, LDL and CRP) in a) maternal plasma, b) cord plasma. Data are presented as beta and 95% confidence intervals. Models included the following variables: maternal age, parity, weight gain during pregnancy, maternal education level, gestational week, and Cd and Pb in maternal blood. Cd and Pb in cord blood were included only in models for cord blood. The smoking status was not include due to the strong correlation between the numbers of cigarettes smoked per day and Cd in maternal blood. Skewed data were normalized using logarithmic (ln) transformation before analysis. MB: maternal blood; CB: umbilical cord blood; HDL: high-density lipoprotein; LDL: low-density lipoprotein; CRP: C-reactive protein. The model for CRP in cord plasma was not tested because the CRP values in cord plasma were below the LOD (LOD = 0.50 mg/L).

Figures 3 and 4 show the results of multiple regression analysis. We estimated the influence of predictors (maternal age, parity, weight gain during pregnancy, education level, gestational week, placental weight, and Cd and Pb in maternal blood, placenta and cord blood) on relative expressions of candidate microRNAs and biochemical parameters in mother-newborn pairs. The Cd in maternal blood was a significant predictor of increased miR-16 (*p* = 0.009) and decreased miR-1537 (*p* = 0.010) and miR-21 (*p* = 0.011) in maternal plasma. Although not statistically significant, Cd in cord blood was a predictor of increased miR-16 (*p* = 0.066) and miR-146a (*p* = 0.105) in cord plasma, while Pb in maternal blood was positively associated with miR-16 in maternal plasma (*p* = 0.077), and negatively associated with miR-16 (*p* = 0.050) and miR-21 (*p* = 0.070) in the placenta. Furthermore, a positive association was also found between gestational week and miR-146a in cord plasma (*p* = 0.001), while parity and miR-190b were negatively associated (*p* = 0.042).

The results of multiple regression analysis for biochemical parameters showed that maternal age was a significant predictor of increased total cholesterol (*p =* 0.009), as well as HDL cholesterol (*p =* 0.012) in maternal plasma. Education was positively associated with total cholesterol (*p* = 0.011) and LDL cholesterol (*p* = 0.030) in maternal plasma. Furthermore, a significant positive association was found between Pb in cord blood and LDL cholesterol in cord plasma (*p* = 0.049), while Cd in maternal blood was positively, though not significantly, associated with total cholesterol (p = 0.088) and triglycerides (p = 0.065) in cord plasma.

We also estimated the influence of different predictors (maternal lifestyle and socioeconomic status, miRNA expression, metal exposure and biochemical parameters) on birth outcomes, birth weight and length (Table 8). Our results showed that miR-146a and urate concentration in maternal plasma and triglycerides in cord plasma were significant predictors of increased birth weight, while miR-21 and miR-16 in maternal plasma and Cd in maternal blood were significant predictors of decreased birth weight. Although the results of forward stepwise multiple regression analysis showed that miRNA expression, toxic metals and biochemical parameters are not significant predictors of birth length, they were retained in the explanatory model indicating that they may contribute to the prediction of birth outcomes.

**Table 8:** Results of forward stepwise multiple linear regression analysis for birth weight and length.∗

Dependent Variable	Equation	Adj.R^2^	*p*
Birth weight	*=****4.1 placental weight +121 gestational week + 374 ln(miR-146a_MP) + 4.9 urate_MP - 161 ln(Cd_MB) - 4.2 urate_CP - 190 ln(miR-21_MP) + 176 gender + 18.4 weight gain - 160 ln(miR-16_MP) + 255 ln(triglycerides_CP)****- 127 ln(miR-146a_CP) - 114 ln(miR-16_CP) + 91 ln(Cd_CB) + 51 ln(miR-1537_MP) +41 ln(miR-1537_CP) + 124 ln(Pb_CB) - 190 ln(T. Chol_CP) + 52 ln(miR-21_PL) - 2188*	0.82	<0.001
Birth length	*=****0.01 placental weight + 0.68 gestational week****- 0.54 ln(miR-146a_CP) + 0.19 ln(miR-1537_MP) + 0.65 ln(Pb_MB) + 0.31 ln(miR-146a_MP) – 0.20 triglycerides_MP + 0.38 gender + 19*	0.60	<0.001

MP: maternal plasma; MB: maternal blood; PL: placenta; CP: umbilical cord plasma; CB: umbilical cord blood; T. Chol: Total cholesterol.

∗ Models included the following variables: maternal age (years); parity; weight gain during pregnancy (kg); gestational week; placental weight (g); gender; relative expression (2^(−ΔCt)^) of all analysed miRNAs; Cd and Pb in maternal blood (μg/L), placenta (μg/kg) and cord blood (μg/L); glucose (mmol/L), total cholesterol (mmol/L), triglycerides (mmol/L) and urate (μmol/L) in maternal and cord plasma. The smoking status was not include due to the strong correlation between the numbers of cigarettes smoked per day and Cd in maternal blood. Skewed data were transformed by logarithmic (ln) before analysis.

As we found the association between miRNAs (miR-146a, miR-21 and miR-16 in maternal plasma) and birth weight, and knowing that smokers have higher expression of miR-16 in maternal and miR-146a in cord plasma and lower miR-21 in placenta, we focused on these associations. In further analyses, we divided the subjects into two subgroups according to overall median: expression > median *vs*. expression ≤ median and we tested the association in both groups, smokers and non-smokers (Table 9). We found that placental weight was a significant predictor of increased birth weight in both group of subjects, non-smokers and smokers, while higher miR-21 in placenta was a significant predictor of increased birth weight only in non-smokers (*p* = 0.007). This association was not found in smokers. We found no association between birth weight and biochemical parameters when we divided the subjects according to their reference values (data not shown).

**Table 9:** Results of multiple linear regression analysis for birth weight.∗

	Intercept	Maternal age	Parity	Weight gain	Gestational week	Placental weight	Male gender	High miR-16_MP	High miR-16_CP	High miR-16_PL	High miR-146a_MP	High miR-146a_CP	High miR-146a_PL	High miR-21_MP	High miR-21_CP	High miR-21_PL
**Non-smokers**																
**F(15, 10) = 6.46; Adj. R**^**2**^**= 0.766; *p* = 0.002**																
Birth weight	-4559 (3669) *p = 0.242*	15 (19) *p = 0.444*	-61 (68) *p = 0.391*	11 (14) *p = 0.442*	148 (104) *p = 0.186*	4.4 (1.11) *p = 0.003*	72 (195) *p = 0.720*	-60 (214) *p = 0.785*	-254 (151) *p = 0.123*	-215 (248) *p = 0.407*	-59 (261) *p = 0.825*	-168 (269) *p = 0.545*	-103 (281) *p = 0.720*	188 (159) *p = 0.264*	-56 (178) *p = 0.761*	652 (195) *p = 0.007*
**Smokers**																
**F(15, 19) = 4.57; Adj. R**^**2**^**= 0.612; *p* = 0.001**																
	-5441 (2505) *p = 0.043*	3.7 (15) *p = 0.809*	4.9 (50) *p = 0.922*	24 (16) *p = 0.154*	170 (68) ***p = 0.022***	3.8 (0.92) ***p = 0.001***	150 (136) *p = 0.283*	253 (196) *p = 0.211*	-258 (166) *p = 0.137*	194 (263) *p = 0.470*	152 (205) *p = 0.469*	-289 (186) *p = 0.135*	-146 (221) *p = 0.516*	89 (195) *p = 0.654*	136 (163) *p = 0.413*	-168 (232) *p = 0.477*

MP: maternal plasma; PL: placenta; CP: umbilical cord plasma.

∗ Models included the following variables: maternal age (years); parity; weight gain during pregnancy (kg); gestational week; placental weight (g); gender; expression of miR-16, miR-146a and miR-21 in maternal plasma, placenta and cord plasma (>median *vs*. ≤median).

## 4 Discussion

### 4.1 Association of maternal smoking and Cd and Pb levels

Maternal cigarette smoking is a matter of great concern in public health due to possible complications during pregnancy and later on in the offspring. In this study, we assessed the effect of maternal smoking on Cd and Pb levels, expression of candidate miRNAs and biochemical parameters in compartments of the maternal-placental-fetal unit: maternal plasma, placenta, and cord plasma. Cigarette smoke contains many harmful chemicals, including toxic metals, mostly Cd and Pb. The half-life of these elements in the body is very long; Cd accumulates in the liver and kidney where it can be retained for many years, while Pb accumulates in bones over one's lifetime [10, 37]. It is well-known that both metals have adverse effects on human health, and this effect is especially pronounced during the perinatal period (pregnancy and lactation) due to specific physiological changes when the absorption and retention of these toxic metals are higher [15, 38].

We found higher concentrations of Cd in all compartments of the maternal-placental-fetal unit in smokers than non-smokers which is in agreement with previous studies, including our own [16, 17, 18, 39, 40]. In the present study, Cd levels in placenta were 13-fold higher in smokers and 21-fold higher in non-smokers than maternal blood levels, and 250-fold and 350-fold higher in smokers and non-smokers, respectively, than cord blood levels. Furthermore, Cd levels in cord blood were only 5–6% of the measured values in maternal blood. Therefore, very low levels of Cd (<0.1 μg/L) could be detected in cord blood and these values were significantly higher in smokers than in non-smokers. These findings indicated that Cd accumulates in the placenta and only a small fraction of Cd passes the placental barrier, which corroborates previous findings [16, 18, 41]. We also found higher levels of Pb in maternal blood and placenta of smokers in comparison to non-smokers, while differences between smokers and non-smokers were not observed for Pb levels in cord blood. Unlike Cd, Pb levels measured in cord blood in this study were 69–76% of the levels in maternal blood pointing to the fact that the placenta is a poor barrier for its transfer to the fetus *in utero* [42, 43] and this transfer is probably a result of passive diffusion [44]*.*

### 4.2 Association of maternal smoking and miRNA expression

In the literature, there is a lack of sufficient information on how maternal smoking affects the miRNA expression in vulnerable population groups such as mother-newborn pairs, and most of the studies investigate the effect of smoking on miRNA expression in the placenta [20, 21]. Encouraged by this, we studied the influence of maternal cigarette smoking on circulating and placental miRNA expression simultaneously using maternal plasma, placenta and umbilical cord plasma. For this purpose, we investigated five miRNAs, which we found in the literature to be associated with maternal smoking and/or toxic metals (Cd and Pb): miR-1537, miR-190b, miR-146a [21], miR-16, and miR-21 [20].

It is known that miRNAs are important for placental development and may have a role in pregnancy loss, diabetes mellitus, intrauterine growth restriction [45]. miRNA-21 expression is detectible in the early stages of embryonal development and it seems that it is involved in the regulation of pluripotency. Some investigations showed that miRNA downregulation in placental tissues decreases migration and cell growth [46]. It is also assumed that miRNA-16 can regulate the Vascular Endothelial Growth Factor (VEGF) gene, which is a very important protein involved in vasculogenesis in the placenta [47]. Furthermore, less is known about the mechanism by which smoking during pregnancy affects fetal development and some suggest that this mechanism may involve miRNAs [4, 20, 48]. When investigating the association between maternal smoking and miRNA expression during pregnancy, it is very important to analyze all compartments (maternal, cord and placenta) of the feto-placental unit to obtain a complete picture, since it is well-known that different sources of miRNAs may influence results and such studies are scarce. Furthermore, changes in miRNA expression obtained from different sources can help in understanding the etiology of the possible impact on pregnancy outcomes [49]. It is known that environmental pollutants can induce changes in miRNA expression and a response to these changes can be the upregulation of DNA and altered gene expression [50]. Brook et al. [51] reported a dysregulation of miRNAs which targets transforming growth factor beta when the placenta was treated with Cd. Also, there is evidence that blood Pb levels in Mexican pregnant women were associated with altered miRNA expression [52]. One of the earliest studies conducted on placentas [20] reported the downregulation of miR-16, miR-21 and miR-146a in placenta of smokers, which is in agreement with our findings of lower expression of miR-21 in placenta of smokers *vs*. non-smokers. The same authors also studied the effects of different concentrations of nicotine and benzo(a)pyrene on the expression of miR-146a in placental cell lines. It was found that nicotine and benzo(a)pyrene caused the downregulation of miR-146a only, and the authors assumed that other components in cigarette smoke or exposure to a total mix of chemicals in smoke were associated with the downregulation of miR-16 and miR-21. It is known that cigarette smoke contains more than a thousand chemicals and many of them are carcinogens [53]. Many components of cigarette smoke, other than nicotine and benzo(a)pyrene, may dysregulate miRNA expression and may have influence on cell growth and development in the placenta, and preterm delivery induced by inflammation [1, 20, 54]. Furthermore, Maccani et al. [20] reported the results of ‘target prediction strategy’ obtained by three algorithms, which they used to predict targets for miR-16, miR-21 and miR-146a. They reported that chemicals from cigarette smoke are associated with the downregulation of miR-21 that may lead to the overproduction of pleomorphic adenoma gene 1 (PLAG1) and special AT-rich sequence-binding protein-1 (SATB1), which are transcription factors involved in cell cycle and production. Also, downregulation of miR-16 by cigarette smoke may lead to the upregulation of ectodysplasin A (EDA), genes involved in the activation of the NFκβ-signaling pathway. In addition, to obtain more information about the effects of different components of cigarette smoke or their mixture on miRNA expression, further analysis *in vivo* and *in vitro* must be conducted on different types of cells. Although we did not find statistically significant differences in the expression between smokers and non-smokers for miR-16 and miR-146a in the placenta, results show a somewhat lower expression of these two miRNAs in smokers *vs*. non-smokers (median 16.8 *vs*. 26.3 for miR-16 and 0.272 *vs*. 0.362 for miR-146a, respectively). However, our results of multiple regression analysis showed that Pb in maternal blood was a predictor of decreased miR-16 in the placenta. Furthermore, studies conducted on human placenta from the National Children's Study (NCS) reported a positive association between high placental Cd and miR-1537 expression, and a negative association between high placental Pb and miR-146a and miR-190b [21]. Our results have shown that there are associations between maternal smoking and/or levels of Cd and Pb and aberrant miRNA expression. We did not find significant correlations between placental Pb and Cd levels and these miRNAs in placenta, but we did find a negative correlation between placental Cd and miR-21 in maternal and miR-190b in cord plasma and a positive correlation between miR-16 in maternal plasma and both placental Cd and Pb. We found that Cd in maternal blood was negatively correlated with miR-1537 in maternal plasma and miR-190b in cord plasma. In addition, positive correlations were found between Cd and Pb in all analyzed compartments and miR-16 in maternal plasma. These disagreements between the studies may be due to a different approach to statistical analysis. We analyzed the association on the entire study group (n = 72), while in the NCS study subjects were dichotomized to equal or less than the median value and greater than the median value. Our results showed lower miR-21 expression in smokers *vs*. non-smokers, and negative correlation between maternal smoking (expressed as the number of cigarettes smoked per day) and miR-21 in placenta. This might lead to the conclusion that downregulating may affect embryonal and fetal development as well as be one of the factors that could affect child and maternal health later in life.

A recent study that investigated associations between maternal pre-pregnancy body mass index (BMI) and seven miRNAs in placenta, including miR-16, miR-21 and miR-146a, found an inverse association between miR-146a in mothers and gestational weight gain below 14 kg [55]. However, we found no significant association of weight gain during pregnancy with the expression of analyzed miRNAs in mother-newborn pairs. There is also evidence in the literature that the expression of miRNAs in the placenta is associated with infant outcomes. Maccani et al. [56] reported that high expression of miR-16 in the placenta was negatively associated with attention score, while high expression of miR-146a in the placenta was positively associated with movement score. Our results of forward stepwise multiple linear regression analysis also showed an association between expression of miR-146a, miR-21 and miR-16 and birth weight, but further analysis, where we focused only on the expression of significant miRNAs, we found that high expression of miR-21 in the placenta was associated with increased birth weight in non-smokers. Furthermore, this association in smokers, even though not significant was negative indicating that smoking-induced alterations in miR-21 may be associated with birth outcomes. Expression of miRNAs in the placenta was also investigated in association with air pollutants. The study conducted on placentas from the ENVIRONAGE birth cohort reported a positive association between exposure to air pollutants during the first trimester and miR-21, while negative associations were reported for exposure during the second trimester and miR-21, miR-146a and miR-222 [55].

In contrast to the studies that included placental tissue, we found only one study that investigated the influence of cigarette smoking on the expression of miRNAs in maternal and cord blood. The reason for this may be the very low amounts of circulating miRNAs in human plasma in relation to the very high expression in placenta, which is also apparent from the results presented in this study. Furthermore, placental samples are relatively easy to collect, which makes them an ideal biological sample for studying the influence of numerous environmental factors on its functions and structure, which may affect fetal development. The placenta is a valuable temporary organ, which provides essential nutrients for normal fetal growth and development, but also acts as a barrier against different xenobiotic agents, including metal(loid)s [15].

In this study, we reported for the first time the association between maternal smoking during pregnancy and the expression of candidate circulating miRNAs, as similar data have not been published so far. We found that the expression of miR-16 in maternal and miR-146a in umbilical cord plasma was significantly associated with maternal smoking during pregnancy, while for other miRNAs we did not find a significant association with smoking. The only available data in the literature discuss the expression of miR-223 [48], which was not analyzed in this study. The authors found that a high concentration of cotinine in urine (as a measure of maternal smoking) was associated with miR-223 in maternal and cord blood. Although there is a lack of literature data on the effect of smoking on the expression of circulating miRNAs, the existing evidence have so far shown that air pollutants [57], working conditions [58, 59] and metal exposure [60] might affect the regulation of circulating miRNA.

### 4.3 Association of maternal smoking and biochemical parameters

Previous studies in the general population of both genders showed that cigarette smoking adversely affects the concentrations of plasma lipids and hematological parameters. Smokers have increased levels of hemoglobin, hematocrit and total leukocyte count [23], triglycerides [23, 61, 62, 63], total cholesterol and LDL levels [22, 23, 62, 63, 64] and reduced levels of HDL [23, 62, 64] in comparison to non-smokers. Although there is increasing evidence that cigarette smoking during pregnancy is the leading cause of adverse maternal and fetal outcomes and causes a variety of lasting ill effects in offspring [24, 65], data on the effect of smoking on metabolic markers during pregnancy have so far been limited. In contrast to the results for the general population, in this study we found no differences in the concentrations of the analyzed biochemical parameters, that is, glucose, triglycerides, total cholesterol, HDL and, LDL levels, between smokers and non-smokers, which corroborates previously reported results [28]. As previously mentioned, numerous studies have confirmed that exposure to cigarette smoke triggers inflammation mechanisms in the human body. The most commonly investigated nonspecific systemic marker of inflammation is CRP, which is elevated in pregnant women due to the maternal inflammatory reaction to the pregnancy [66, 67, 68]. In addition to the body's natural reaction to pregnancy, unhealthy lifestyle habits, such as cigarette smoking, are also associated with elevated CRP levels [69]. However, we found no difference in CRP levels between smokers and non-smokers in this study, which is in accordance with the results of Zagożdżon et al. [22] for women of reproductive age.

Various clinical and experimental studies investigating the effect of smoking on plasma urate content have yielded conflicting results. A study by Lain et al. [28], who investigated the influence of smoking on urate levels and other markers during normal pregnancy, showed that urate levels were significantly higher in smokers than in non-smokers. Kim et al. [29, 30] also found higher uric levels in female smokers of reproductive age in comparison to non-smokers. However, in our study concentrations of urate in maternal plasma of smokers were lower than in non-smokers. Our observations are consistent with findings by Mouhamed et al. [27] for the general population, who also found significantly lower plasma urate concentrations in smokers of both genders. This could be expected as urate is a powerful scavenger of free radicals, which provides ∼60% of free-radical scavenging capacity in plasma [70]. It is consumed in situations of increased exposure to oxidative stress, including smoking [27, 71]. In addition, lower urate content in smokers' plasma can be attributed to a reduction of the endogenous production of urate as a result of chronic exposure to cigarette smoke [27]. The reasons for the conflicting findings on the effect of smoking on serum urate levels are unclear, but these differences could be related to inconsistent definitions of smoking exposure based on self-reporting [30].

In this study, results obtained in maternal and cord plasma of smokers and non-smokers for triglycerides, urate, total as well as HDL and LDL cholesterol were in good agreement with the reference intervals reported for healthy neonates aged 1–3 days [72] and for maternal and cord blood [73]. Glucose levels in cord plasma were 75% of levels measured in maternal plasma of both smokers and non-smokers, indicating free transport from maternal blood. Glucose has an important role in insulin secretion in fetuses which is important for normal fetal growth [74]. Unlike glucose, lipids such as LDL, HDL and total cholesterol are transferred from mother to fetus in small quantities [75]. In addition, triglycerides are not transported from maternal blood; they are produced by the fetal liver and released in circulation subsequent to the fatty acid esterification [76].

### 4.4 Study limitation

When interpreting the results obtained in this study, caution is required due to a few limitations. Firstly, the relatively small number of participants (n = 72) which can influence on results to identify miRNA biomarkers [77], although other studies had an even smaller number of subjects [20, 21]. In addition, the small sample size also means significantly reduced statistical power that may lead to the detection of false positive findings, which is drastically reduced in studies with a large number of subjects. Secondly, relatively small panel of analyzed candidate miRNAs. In order to select miRNAs candidate for analysis, high-throughput screening is performed, but this was too expensive. Therefore, we investigated candidate miRNAs that we found in the literature to be associated with smoking. Thirdly, the cohort characteristics, definition of inclusion and exclusion criteria across the studiers makes it difficult to compare the obtained results leading to inconsistencies among the studies [78]. Fourthly, methods of sampling, especially for the placenta; due to different miRNA expressions, some miRNAs are highly expressed in villous trophoblasts than in the extravillous trophoblasts [79]. It is also necessary to establish standardized methodologies for placenta sampling to improve reproducibility.

## 5 Conclusions

We confirmed previous findings that maternal smoking increased levels of toxic metals (Cd and Pb) in all compartments of the feto-placental unit. We also reported that maternal smoking was associated with decreased urate concentrations, indicating that urate, as a powerful scavenger of free radicals, neutralized free radicals from cigarette smoke. This study is the first to report results of the expression of cellular and circulating miRNAs simultaneously in maternal and fetal compartments and in the placenta. This study also showed that the expression of circulating candidate miRNAs in maternal and cord blood plasma may be related with exposure to Cd and Pb due to maternal smoking. Our findings propose that miRNAs have the potential to serve as early markers of the developmental origins of health and disease, but further investigations are needed with a larger number of participants and analyzed miRNAs with well-defined specific characteristics of the studied population group, type of exposed cells, duration and source of exposure and other factors that may have impact on miRNA regulation.

## Declarations

### Author contribution statement

Ankica Sekovanić, Tatjana Orct, Zorana Kljaković-Gašpić: Performed the experiments; Analyzed and interpreted the data; Wrote the paper.

Adrijana Dorotić: Performed the experiments; Analyzed and interpreted the data; Contributed reagents; materials, analysis tools or data; Wrote the paper.

Daria Pašalić: Conceived and designed the experiments; Performed the experiments; Analyzed and interpreted the data; Contributed reagents; materials; analysis tools or data; Wrote the paper.

Antonija Sulimanec Grgec: Performed the experiments.

Sandra Stasenko, Tatjana Mioč: Contributed reagents, materials, analysis tools or data.

Martina Piasek, Jasna Jurasović: Conceived and designed the experiments; Contributed reagents, materials, analysis tools or data; Wrote the paper.

### Funding statement

The work was supported by the Croatian Science Foundation during research project “Assessment of Daily Exposure to Metals and Maternal Individual Susceptibility as Factors of Developmental Origins of Health and Disease, METALORIGINS” (grant HRZZ-IP-2016-06-1998).

### Data availability statement

The data that support the findings of this study are available from the corresponding author, upon reasonable request.

### Declaration of interest's statement

The authors declare no competing interests.

### Additional information

No additional information is available for this paper.

## Appendix A Supplementary data

Supplementary Materials Table S1-S3_Sekovanic et al.docx Supplementary Materials Table S1-S3_Sekovanic et al.docx
