# Supplementary material for: The effects of maternal cigarette smoking on cadmium and lead levels, miRNA expression and biochemical parameters across the feto-placental unit
Source: Heliyon. 2022 Dec 24;8(12):e12568. doi: 10.1016/j.heliyon.2022.e12568 (PMC9830161; doi:10.1016/j.heliyon.2022.e12568)
Supplement: Supplementary Materials Table S1-S3_Sekovanic et al.docx [file mmc1.docx]

**Supplementary materials**

# Journal: Heliyon

Title: The effects of maternal cigarette smoking on cadmium and lead levels, miRNA expression and biochemical parameters across the feto-placental unit

Authors: Ankica Sekovanić, Adrijana Dorotić, Daria Pašalić, Tatjana Orct, Zorana Kljaković-Gašpić, Antonija Sulimanec Grgec, Sandra Stasenko, Tatjana Mioč, Martina Piasek and Jasna Jurasović

Corresponding author:

Daria Pašalić

Department of Medical Chemistry, Biochemistry and Clinical Chemistry, University of Zagreb, School of Medicine, Zagreb, Croatia

e-mail: daria.pasalic@mef.hr

Supplementary material contains 3 Tables

**Table S1** UltraCLAVE IV (Milestone, Sorisole, Italy) temperature program used for placenta digestion

|  | **T (min:s)** | **E (W)** | **T (°C)** | **P (bar)** |
| --- | --- | --- | --- | --- |
| 1. | 5 | 1000 | 80 | 100 |
| 2. | 10 | 500 | 130 | 100 |
| 3. | 4:30 | 1000 | 180 | 120 |
| 4. | 6:30 | 1000 | 220 | 130 |
| 5. | 20 | 1000 | 220 | 130 |

**Table S2** ICP-MS Agilent 7500cx (Agilent Technologies, Tokyo, Japan) working conditions

| **Parameter** |  |  | |  |
| --- | --- | --- | --- | --- |
| RF Power | | 1550 W | |  |
| RF matching | | 1.68 V | |  |
| Sampling depth | | 7.5 mm | |  |
| Torch-H | | 0.4 mm | |  |
| Torch-V | | -0.7 mm | |  |
| Nebulizer pump | | 0.08 rps | |  |
| Plasma gas flow rate | | 15 L/min | |  |
| Makeup gas flow rate | | 0.1 L/min | |  |
| Carrier gas flow rate | | 1.05 L/min | |  |
| Nebulizer | | MicroMist (quartz) | |  |
| Spray chamber | | Scott type (quartz), cooled at 2^o^C | |  |
| Sample cone | | Nickel, 1 mm orifice diameter | |  |
| Skimmer cone | | Nickel, 0.4 mm orifice diameter | |  |
| Doubly-charged ions and oxides limits | | ^140^Ce^2+^/^140^Ce^+^<1.0 %; ^140^Ce^16^O^+^/^140^Ce^+^<1.3 % | |  |
|  | | | **He** | |
| Collision/reaction gas flow rate | | | 4.2 mL/min | |
| Extract lens 1 voltage | | | 0.5 V | |
| Extract lens 2 voltage | | | -150 V | |
| Isotopes measured | | | ^114^Cd, ^208^Pb, | |

**Table S3** List of methods used in biochemical analysis

| **Analyte** | **Method** |
| --- | --- |
| Total cholesterol | Enzymatic method with esterase, cholesterol oxidase and peroxidase |
| HDL cholesterol | Enzymatic method with elimination chylomicrons, VLDL and LDL lipoproteins |
| LDL cholesterol | Enzymatic method with elimination all non-LDL lipoproteins |
| Glucose | Enzymatic method with hexokinase |
| Urate | Enzymatic method with uricase and peroxidase |
| Triglyceride | Enzymatic method with glycerol-phosphate oxidase and peroxidase |
| CRP | Immunoturbidimetric method |
